# Supplementary material for: Regulatory RNA Networks in Ovarian Follicular Cysts in Dairy Cows: Implications for Human Polycystic Ovary Syndrome
Source: Genes (Basel). 2025 Jun 30;16(7):791. doi: 10.3390/genes16070791 (PMC12294580; doi:10.3390/genes16070791)
Supplement: Supplementary file 1 [file genes-16-00791-s001.zip › TableS1.pdf]

Table S1. miRBase profiler, consisting of cow-specific primers for 84 target miRNAs and primers for control genes.

	1	2	3	4	5	6	7	8	9	10	11	12
**A**	bta-let-7f	bta-miR-101	bta-miR-103	bta-miR-125a	bta-miR-125b	bta-miR-126-3p	bta-miR-128	bta-miR-145	bta-miR-148a	bta-miR-151-3p	bta-miR-151-5p	bta-miR-16b
**B**	bta-miR-181a	bta-miR-18a	bta-miR-18b	bta-miR-199a-5p	bta-miR-205	bta-miR-20a	bta-miR-21-5p	bta-miR-221	bta-miR-222	bta-miR-26a	bta-miR-26b	bta-miR-27a-3p
**C**	bta-miR-27b	bta-miR-29a	bta-miR-30b-5p	bta-miR-30d	bta-miR-31	bta-miR-320a	bta-miR-34b	bta-miR-484	bta-miR-499	bta-miR-99a-5p	bta-let-7a-5p	bta-let-7d
**D**	bta-let-7g	bta-let-7i	bta-miR-106a	bta-miR-107	bta-miR-10a	bta-miR-10b	bta-miR-122	bta-miR-124a	bta-miR-127	bta-miR-132	bta-miR-138	bta-miR-139
**E**	bta-miR-140	bta-miR-142-3p	bta-miR-142-5p	bta-miR-148b	bta-miR-150	bta-miR-15b	bta-miR-17-3p	bta-miR-17-5p	bta-miR-181b	bta-miR-181c	bta-miR-186	bta-miR-191
**F**	bta-miR-192	bta-miR-193a-3p	bta-miR-193a-5p	bta-miR-199a-3p	bta-miR-199b	bta-miR-200a	bta-miR-200b	bta-miR-200c	bta-miR-20b	bta-miR-210	bta-miR-21-3p	bta-miR-214
**G**	bta-miR-215	bta-miR-218	bta-miR-22-5p	bta-miR-23a	bta-miR-23b-3p	bta-miR-24-3p	bta-miR-25	bta-miR-29b	bta-miR-29c	bta-miR-30a-5p	bta-miR-30c	bta-miR-30e-5p
**H**	cel-miR-39-3p	cel-miR-39-3p	SNORD 42B	SNORD 69	SNORD 61	SNORD 68	SNORD 96A	RNU6-6P	miRTC	miRTC	PPC	PPC
