# Supplementary material for: Regulatory RNA Networks in Ovarian Follicular Cysts in Dairy Cows: Implications for Human Polycystic Ovary Syndrome
Source: Genes (Basel). 2025 Jun 30;16(7):791. doi: 10.3390/genes16070791 (PMC12294580; doi:10.3390/genes16070791)
Supplement: Supplementary file 1 [file genes-16-00791-s001.zip › TableS2.pdf]

Table S2. Bovine and human sequences for dysregulated miRNAs

<u>Bovine and human sequences for upregulated miRNAs</u>

**Bovine**		**Human**	
**miRNA**	**Sequence**	**miRNA**	**Sequence**
bta-mir-132	UAACAGUCUACAGCCAUGGUCG	UAACAGUCUACAGCCAUGGUCG	hsa-mir-132-3p
bta-mir-21-3p	AACA**G**CAGUCGAUGGGCUGU**CU**	CAACA**C**CAGUCGAUGGGCUGU	hsa-mir-21-3p
bta-mir-199b	CCCAGUGUUUAGACUAUCUGUUC	CCCAGUGUUUAGACUAUCUGUUC	hsa-mir-199b-5p
bta-mir-103	AGCAGCAUUGUACAGGGCUAUGA	AGCAGCAUUGUACAGGGCUAUGA	hsa-mir-103a-3p
bta-mir-221	AGCUACAUUGUCUGCUGGGUUU	AGCUACAUUGUCUGCUGGGUUUC	hsa-mir-221-3p
bta-mir-101	UACAGUACUGUGAUAACUGAA	UACAGUACUGUGAUAACUGAA	hsa-mir-101-3p
bta-mir-99a-5p	AACCCGUAGAUCCGAUCUUGU	AACCCGUAGAUCCGAUCUUGU**G**	hsa-mir-99a-5p
bta-mir-145	GUCCAGUUUUCCCAGGAAUCCCU	GUCCAGUUUUCCCAGGAAUCCCU	hsa-mir-145-5p
bta-mir-193a-3p	AACUGGCCUACAAAGUCCCAGU	AACUGGCCUACAAAGUCCCAGU	hsa-mir-193a-3p
bta-mir-29c	UAGCACCAUUUGAAAUCGGUUA	UAGCACCAUUUGAAAUCGGUUA	hsa-mir-29c-3p

Red-colored nucleotide indicates the difference between human and bovine sequences

<u>Bovine and human sequences for downregulated miRNAs</u>

**Bovine**		**Human**	
**miRNA**	**Sequence**	**miRNA**	**Sequence**
bta-mir-18a	UAAGGUGCAUCUAGUGCAGAUA	UAAGGUGCAUCUAGUGCAGAUA**G**	hsa-mir-18a-5p
bta-mir-30e-5p	UGUAAACAUCCUUGACUGGAAG**CU**	UGUAAACAUCCUUGACUGGAAG	hsa-mir-30e-5p
bta-mir-30b-5p	UGUAAACAUCCUACACUCAGCU	UGUAAACAUCCUACACUCAGCU	hsa-mir-30b-5p
bta-mir-15b	UAGCAGCACAUCAUGGUUUACA	UAGCAGCACAUCAUGGUUUACA	hsa-mir-15b-5p
bta-mir-29a	**C**UAGCACCAUCUGAAAUCGGUUA	UAGCACCAUCUGAAAUCGGUUA	hsa-mir-29a-3p
bta-mir-26b	UUCAAGUAAUUCAGGAUAGGU**U**	UUCAAGUAAUUCAGGAUAGGU	hsa-mir-26b-5p
bta-mir-191	CAACGGAAUCCCAAAAGCAGCUG	CAACGGAAUCCCAAAAGCAGCUG	hsa-mir-191-5p

Red-colored nucleotide indicates the difference between human and bovine sequences
