# Supplementary material for: Regulatory RNA Networks in Ovarian Follicular Cysts in Dairy Cows: Implications for Human Polycystic Ovary Syndrome
Source: Genes (Basel). 2025 Jun 30;16(7):791. doi: 10.3390/genes16070791 (PMC12294580; doi:10.3390/genes16070791)
Supplement: Supplementary file 1 [file genes-16-00791-s001.zip › TableS3.pdf]

Table S3: Network-Based Identification of Key Genes Influenced by Differentially Expressed miRNAs

Gene	Degree	Betweenness	Granulosa Cell* Protein Expression (nTPM)[§]
Top 15 Genes with High Centrality Metrics Regulated by Upregulated miRNAs			
UBC	85	1962.234	1333.3
RFX3	27	252.5443	7.7
FN1	26	145.8581	9.4
MYC	26	128.6414	104.9
ATXN1	25	118.977	21.5
CUL3	25	118.5346	27.4
ESR1	23	118.1186	6.3
RFX4	22	112	0.2
TNRC6B	20	92.4799	103.5
GRB2	20	83.85947	31.1
ELAVL1	19	83.85032	40.6
CREB1	18	83.77242	28.9
PIK3R1	15	79.24764	85.2
CDK2	15	78.21544	9.7
HSP90AA1	15	65.93539	2468.2
Top 15 Genes with High Centrality Metrics Regulated by Downregulated miRNAs			
UBC	48	530.7089	1333.3
ELAVL1	27	102.6451	40.6
CUL3	24	101.2921	27.4
ESR1	19	70.29211	6.3
MYC	17	59.55803	104.9
CDK6	15	48.57422	25.1
PTEN	14	30.19794	22.8
TNRC6A	14	29.91954	54.3
SERBP1	14	27.36892	199.7
PTP4A1	13	27.34294	0.2
BCL2	13	27.31308	44.1
MCL1	12	24.9079	411.1
CDKN1A	12	24.62622	85.2
THBS1	11	22.78579	60.1
TUBB	11	22.05779	345.3

*Granulosa cells count - 471;

[§]The Human Protein Atlas; https://www.proteinatlas.org/ (accessed on 13 February 2025).

UBC – Ubiquitin C; *RFX3* - Regulatory factor X3; *FN1* – Fibronectin1; *MYC* - MYC proto-oncogene, bHLH transcription factor; *ATXN1* – Ataxin 1; *CUL3* – Cullin 3; *ESR1* - Estrogen receptor 1; *RFX4* - Regulatory factor X4; *TNRC6B* - Trinucleotide repeat containing adaptor 6B; *GRB2* - Growth factor receptor bound protein 2; *ELAVL1* - ELAV like RNA binding protein 1; *CREB1* - CAMP responsive element binding

protein 1; *PIK3R1* - Phosphoinositide-3-kinase regulatory subunit 1; *CDK2* - Cyclin dependent kinase 2; *HSP90AA1* - Heat shock protein 90 alpha family class A member 1; *CDK6* - Cyclin dependent kinase 2; *PTEN* - Phosphatase and tensin homolog; *TNRC6A* - Trinucleotide repeat containing adaptor 6A; *SERBP1* - SERPINE1 mRNA binding protein 1; *PTP4A1* - Protein tyrosine phosphatase 4A1; *BCL2* - BCL2 apoptosis regulator; *MCL1* - MCL1 apoptosis regulator, BCL2 family member; *CDKN1A* - Cyclin dependent kinase inhibitor 1A; *THBS1* - Thrombospondin 1; *TUBB* - Tubulin beta class I;
