# Supplementary figures and images for: Regulatory RNA Networks in Ovarian Follicular Cysts in Dairy Cows: Implications for Human Polycystic Ovary Syndrome
Source: Genes (Basel). 2025 Jun 30;16(7):791. doi: 10.3390/genes16070791 (PMC12294580; doi:10.3390/genes16070791)

Figure S1. Schematic diagram of analysis.

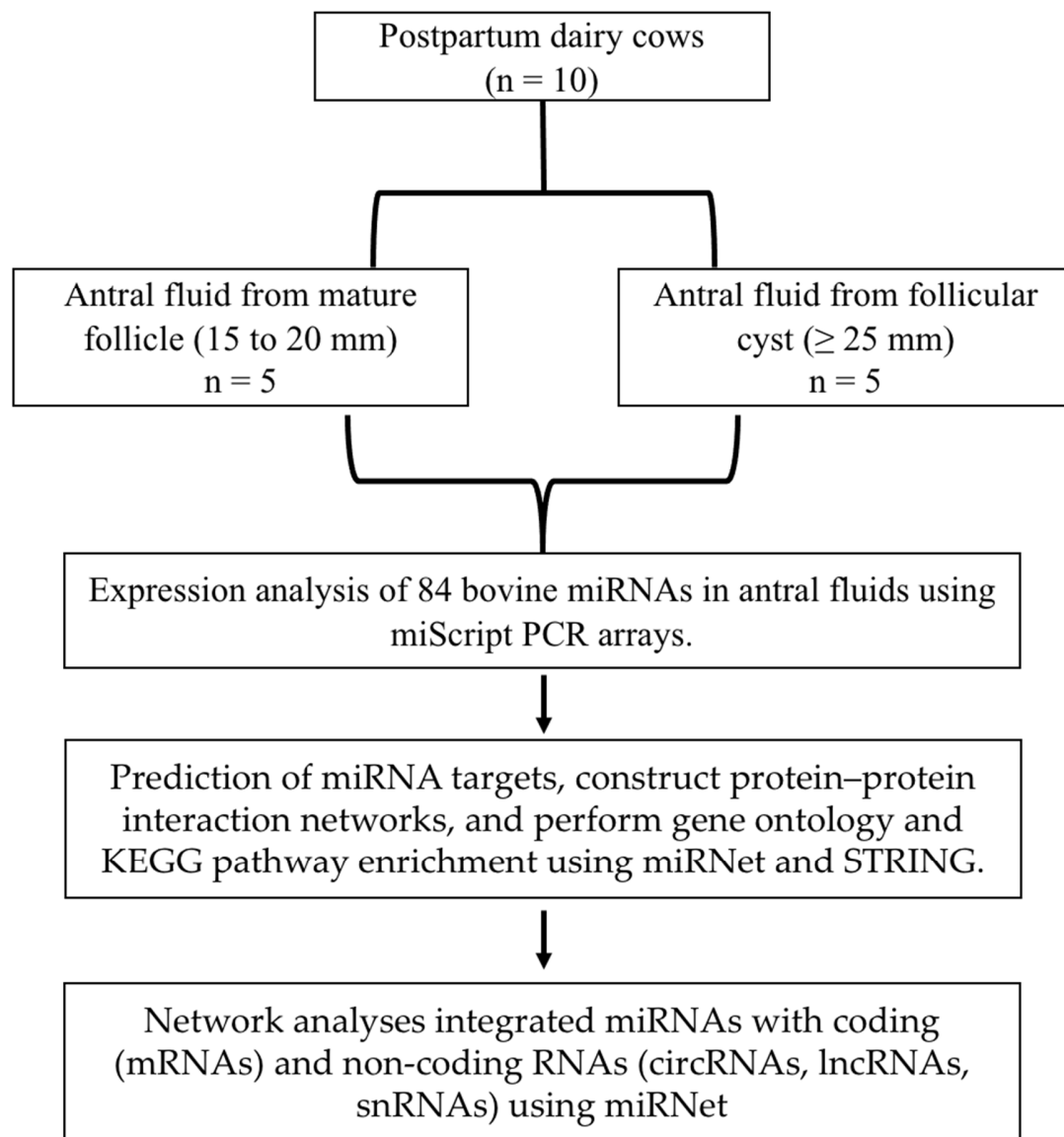

Supplement: Supplementary file 1 [file genes-16-00791-s001.zip › FigureS1.pdf]
